# A *GmRAV* Ortholog Is Involved in Photoperiod and Sucrose Control of Flowering Time in Soybean

**DOI:** 10.1371/journal.pone.0089145

**Published:** 2014-02-14

**Authors:** Qingyao Lu, Lin Zhao, Dongmei Li, Diqiu Hao, Yong Zhan, Wenbin Li

**Affiliations:** 1 Key Laboratory of Soybean Biology of Chinese Education Ministry (Key Laboratory of Biology and Genetics & Breeding for Soybean in Northeast China, Ministry of Agriculture), Northeast Agriculture University, Harbin, China; 2 Agricultural Academy of Shi He Zi, Xinjiang Province, China; Nanjing Agricultural University, China

## Abstract

Photoperiod and sucrose levels play a key role in the control of flowering. *GmRAV* reflected a diurnal rhythm with the highest expression at 4 h after the beginning of a dark period in soybean leaves, and was highly up-regulated under short-day (SD) conditions, despite of not following a diurnal pattern under long-day (LD) conditions. *GmRAV-i* (*GmRAV*-inhibition) transgenic soybean exhibited early flowering phenotype. Two of the *FT* Arabidopsis homologs, *GmFT2a* and *GmFT5a*, were highly expressed in the leaves of soybeans with inhibition (-i) of *GmRAV* under SD conditions. Moreover, the transcript levels of the two *FT* homologs in *GmRAV-i* soybeans were more sensitive to SD conditions than LD conditions compared to the WT plant. *GmRAV*-i soybeans and *Arabidopsis rav* mutants showed more sensitive hypocotyl elongation responses when compared with wild-type seedlings, and *GmRAV*-ox overevpressed in tobacco revealed no sensitive changes in hypocotyl length. These indicated that *GmRAV* was a novel negative regulator of SD-mediated flowering and hypocotyl elongation. Although sucrose has been suggested to promote flowering induction in many plant species, high concentration of sucrose (4% [w/v]) applied into media defer flowering time in Arabidopsis wild-type and *rav* mutant. This delayed flowering stage might be caused by reduction of *LEAFY* expression. Furthermore, Arabidopsis *rav* mutants and *GmRAV*-i soybean plants were less sensitive to sucrose by the inhibition assays of hypocotyls and roots growth. In contrast, transgenic *GmRAV* overexpressing (-ox) tobacco plants displayed more sensitivity to sucrose. In conclusion, *GmRAV* was inferred to have a fundamental function in photoperiod, darkness, and sucrose signaling responses to regulate plant development and flowering induction.

## Introduction

In many plant species, flowering time is strongly influenced by environmental factors where photoperiod plays a prominent role [1]. Plants perceive light through its phytochromes and cryptochromes, which transfer the signal to the plant internal circadian clock system. There is an increasing evidence for conservation of flowering pathways between many plant species. Flowering time is regulated by multiple and to some extent redundant pathways that can promote or delay flowering [2]. *Arabidopsis thaliana*, a model organism, is a long-day plant, and there are many pathways, such us photoperiod, vernalization, gibberellic acid and autonomous reactions, involved in control of its floral transition [3]–[6]. In contrast, soybean (*Glycine max*) is a short-day plant, and photoperiod controls its duration in both pre- and post-flowering phases [7]. Therefore, photoperiod is an important environmental cue that determines flowering time in soybean [8]. The term ‘critical photoperiod’ is described as the duration of daylight period under which the plant is induced to flower, and determines plant transition from vegetative to reproductive stage. Sensitivity to photoperiod limits the adaptation of soybean to a wider range of latitude [9]. The identification of the genetic components contributing to the photoperiodic control of flowering time in soybean was recently limited.

Florigen (FT) is a hypothetical leaf-produced signal that moves from phloem to induce flowering at shoot apex. The expression of *FT* gene (the flowering integrator genes, FLOWERING LOCUS T [10], and its orthologs are critical for flowering in plants [11], [12]. Two soybean *FT* homologs (*GmFT2a* and *GmFT5a*) have florigen-like functions and their transcript levels are upregulated under SD conditions (SDs) [13].

Not only carbohydrates provide energy and carbon sources for plants, but also act as essential regulators during their growth and development [14], [15], as evidenced by the variety of sugar sensing and signaling mechanisms that have been uncovered [16], [17]. Carbohydrates seem to regulate many essential processes, including photosynthesis, sucrose synthesis and degradation, flowering, and senescence [18], [19].

There has been a certain amount of evidence suggesting that sucrose promotes flowering in most species [20]. In Arabidopsis, the induction of flowering in wild-type plants by LDs causes an early and transient increase in sucrose export from leaves. The efficiency of floral induction by a single LD is reflected by the amplitude of an increase in exported sucrose [21]. Rolda’n et al. reported that *in vitro* culture of plants on medium containing 1% (w/v) sucrose, partially rescued the phenotypes of late-flowering mutants [22]. In contrast, Zhou et al. reported that high levels of glucose in the medium delayed flowering in Arabidopsis [23]. Masa-aki et al. also analyzed the effects of sugar on development and floral transition [24]. In an early flowering mutant *tfl1*, 5% (w/v) sucrose in the medium delayed floral transition. It was concluded that the inhibition was caused by metabolic rather than its osmotic effects. Recently, King et al. reported that *FT* and sucrose may regulate flowering as ‘a florigen’ in plants [25].

Despite their metabolic role, glucose and fructose play additional signaling functions in plant cells [26]. Oligosaccharides derived from the cell wall also function as signals in the processes of regulation of hypocotyl elongation [27], fruit ripening [28] and defense mechanisms to pathogens [29]. Also, root growth was considerably more sensitive to carbon source than hypocotyl elongation [30]. In this study, the effects of sugars on a range of growth and developmental parameters in *Arabidopsis thaliana*, tobacco and soybean were measured. The effects of sugars on growth and developmental processes in plants at earlier stages of vegetative development and the flowering timing were also investigated.

In Arabidopsis, the RAV subfamily belongs to one of the largest and most diverse family of transcription factors AP2/ERBP. RAV proteins function in the involvement in cold tolerance, dehydration, and circadian rhythm clock. *GmRAV* (DQ147914) [31], [32] was one of four *RAV2*-like paralogues in the soybean genome. *GmRAV* may be a complete functional orthologue of any *AtRAV2* family member [33].

In this study, *GmRAV* was overexpressed in transgenic tobacco and inhibited in transgenic soybean, which showed that *GmRAV* was a responder in the photoperiodic control of flowering time and sugar signaling. We found that *GmRAV* transcript exhibited a circadian rhythm under SDs and decreased significantly in leaves by exogenous sucrose application. A detailed phenotypic characterization, along with genetic and physiological analysis, indicated that *GmRAV* was inferred to be a signaling component involved in regulation of plant development and flowering time.

## Materials and Methods

### Plant Materials and Growth Conditions

*Arabidopsis thaliana Columbia* (Col-0) ecotype was used in this study as wild-type plant (Lehle Seeds, Round Rock, TX). The Salk T-DNA knockout mutant line of *AtRAV* (At1g25560; SALK_029626c) was obtained from the *Arabidopsis* Biological Resource Center (ABRC). *GmRAV-i* soybean, *GmRAV-ox* tobacco and *GmRAV* promoter::GUS transgenic Arabidopsis seeds were provided by our lab (Northeast Agricultural University, Harbin, China) [33]. The primer pairs for transgenic plants detection were listed in Table 1. Arabidopsis seeds were surface sterilized, placed in Petri dishes containing solid Murashige and Skoog (MS) medium, and stratified for 3 days at 4°C. Subsequently, the seedlings were placed in a vertical orientation in the growth chamber at 22°C under LDs (16 h/8 h light/dark). T4 generation *GmRAV-ox* tobacco and T6 generation transgenic *GmRAV-i* soybean [33] were grown in a growth chamber at 25°C, and illuminated with 200 µmol·m^−2^·s^−1^ fluorescent lights.

**Table 1:** List of primers for transgenic plants detection used in the present study.

Primer name	Primer sequence
*pat*-F (soybean)	GCACCATCGTCAACCACTAC
*pat*-R	TGAAGTCCAGCTGCCAGAAAC
Salk_029626c LP	AATCTCATGTGAACCCCCTTC
Salk_029626c RP	CGCTGATGCTTCTCGTAAATC
Salk_029626c LB	ATTTTGCCGATTTCGGAAC

Seeds of soybean cultivars ‘Dong Nong 42’ and ‘Dong Nong 47’ (provided by Northeast Agricultural University, Harbin, China; photoperiod sensitive) and ‘*GmRAV-i* transgenic soybean’ were grown at 25°C under LDs with 250 µmol·m^−2^·sec^−1^ white light. The plants were transferred to SDs under the same temperature regime after V2 stage. Experiments were conducted under LDs of 16 h/8 h light/dark and SDs of 8 h/16 h light/dark. Seeds of the WT plants and Arabidopsis *rav* mutants were sowed in solid MS medium in Petri dishes, and then were conducted for the same treatments as above.

In diurnal expression analysis, pieces of young fully developed trifoliate leaves were sampled as a bulk of three plants grown under LDs at 15 days after emergence (DAE) every 4 h starting at dawn for a total of 24 h. Also, plant tissues were harvested from root, stem, leaf, trifoliate leaf, flower bud, pod and immature seed at 12 h after dawn under SDs. In time course-dependent expression analysis, the trifoliate leaves from ‘Dong Nong 47’ soybean plants were sampled at 12 h after dawn by bulk from four individual plants grown in SDs and LDs at 17, 20, 23, 26, 29, 32, 35 and 45 DAE. The dates of the first flower appearance and flower bud formation at each node were recorded individually.

### RNA Isolation and Quantitative Real-time RT-PCR (qRT-PCR) Analysis

Total RNA was extracted from soybean and *Arabidopsis* seedlings with RNAiso Plus Kit (TaKaRa, Japan). The total RNA was reverse-transcribed into first-strand cDNA in a 20 µL volume with PrimeScript RT reagent Kit (TaKaRa, Japan). qRT-PCR analysis was carried out using SYBR Premix Ex Taq II (TaKaRa, Japan) in a 25 µL reaction, containing 2 µL of cDNA, 12.5 µL SYBR Premix Ex Taq II (2×), 1 µL of 10 µM forward primer, 1 µL of 10 µM reverse primer and 8.5 µL of water. The reaction was performed in the Thermal Cycler Dice Real Time System. The thermal cycle used was as follows: 95°C for 30 s; 40 cycles of 95°C for 5 s, 60°C for 20 s and 72°C for 20 s. Soybean actin 4 (*GmACTIN*; GenBank accession number AF049106) and Arabidopsis 18 s rRNA (GenBank accession number X16077.1) were included as inner references for soybean and Arabidopsis genes. The total RNA was used as templates in qRT-PCR reactions with the primers of *GmRAV*, *GmFT2a*, *GmFT5a* and *AtLEAFY* genes. The primer pairs were listed in Table 2. PCR reactions were performed according to the manufacturer’s instructions on the Chromo 4 real time DNA amplification system (BioRad, USA). Data were analyzed using the comparative Ct method. Further qRT-PCR analysis were performed as described above. The analysis were done using the DNA Engine Opticon 2 System (MJ Research, USA). The sequences reported in this paper have been deposited in the GenBank/EMBL/DDBJ database with accession numbers AB550122 (*GmFT2a*), AB550126 (*GmFT5a*) for cDNA sequences of soybean cultivar ‘Dong Nong 50’ and Genbank accession number AF010190.2 (*AtLEAFY*) for cDNA sequences of Arabidopsis.

**Table 2:** List of primer for real-time PCR analysis used in the present study.

Primer name	Primer sequence
At18SrRNA-F	CGTCCCTGCCCTTTGTACAC
At18SrRNA-R	CGAACACTTCACCGGATCATT
*AtLEAFY*-F	TGTGAACATCGCTTGTCGTC
*AtLEAFY*-R	TAATACCGCCAACTAAAGCC
*GmACTIN4*-F	GTGTCAGCCATACTGTCCCCATTT
*GmACTIN4*-R	GTTTCAAGCTCTTGCTCGTAATCA
*GmRAV*-F	GGTTCGGATGGTGTAGGGAAGAGAA
*GmRAV*-R	TTACAAAGCTCCAATTACTTTTAAC
*GmFT2a*-F	GGATTGCCAGTTGCTGCTGT
*GmFT2a*-R	GAGTGTGGGAGATTGCCAAT
*GmFT5a*-F	GCCTTACTCCAGCTTATACT
*GmFT5a*-R	GGCATGCTCTAGCATTGCAA

### GUS Assays

*GUS* activity was assayed in T3 transgenic Arabidopsis plants. GUS histochemical staining and GUS activity measurements (using about 40–50 seedlings in each sample) were carried out following the procedures described by Jefferson et al. [34].

### Sucrose Stress Assay

The sucrose sensitivity assay for hypocotyl elongation was carried out by germinating the wild-type and *rav* transgenic seeds. The change in root and hypocotyl length was used as a measure to check the sensitivity of the plants using the Image-J program (http://rsb.info.nih.gov/ij/docs/menus/file.html) after 15 days.

For flowering assay, the wild-type and transgenic seeds were grown on MS medium supplemented with different concentrations of sucrose during their lifetime. Plants were growing on MS with 2% and 4% (w/v) sucrose, and after 15 days were transferred into soil till flowering. Flowering time was measured by scoring the time from sowing to first flower.

### Statistical Analysis

Data was presented as means ± standard error of means. The statistical comparisons were made using Student’s t test at p<0.01 or p<0.05.

## Results

### The Accumulation of GmRAV Transcript is Regulated by Photoperiod and Darkness

**Figure 1:**
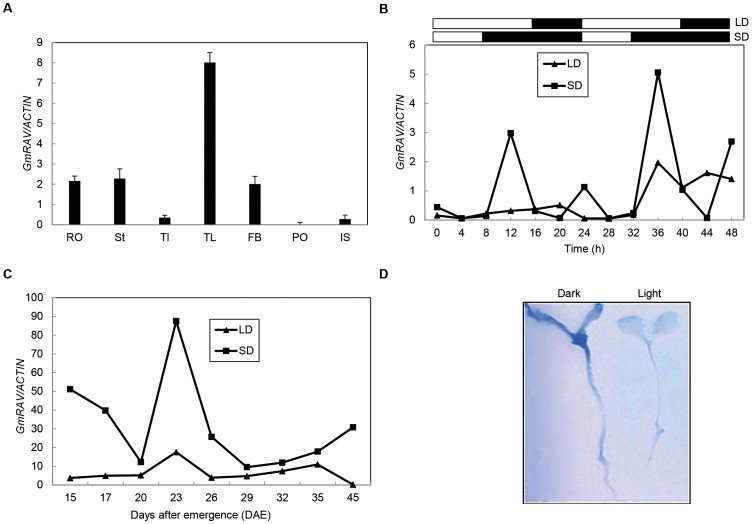
Quantitative real-time RT-PCR analysis of transcript level of *GmRAV* gene under SDs and LDs. A, Tissue-specific expression of soybean *GmRAV* in SDs. Tissues tested are leaf (Tl), trifoliate leaf (TL), stem (St), root (RO), pod (PO), flower bud (FB), and immature seed (IS) (plants aged 21 d). B, Relative transcript levels of *GmRAV* mRNA in soybean leaves under SDs and LDs. Soybean leaves were harvested every 4 h for 48 h at 25-day-old under LDs and SDs. *Open* and *closed boxes* indicate days and nights. C, Time course-dependent expression in LDs. Soybean ‘Dong Nong 47’ plants were grown under LDs for 10 d and were transferred to LDs or SDs before sampling. Relative transcript levels were analyzed by qRT-PCR. D, Histochemical detection of *GmRAV*–GUS promoter activity in transgenic *Arabidopsis* seedlings. 4-day-old seedlings were grown on MS medium.

Transcription profiles of *GmRAV* were analyzed in various tissues of ‘Dong Nong 42’ soybean grown under inductive SDs at 12 h after dawn by quantitative real-time RT-PCR. *GmRAV* mRNA was present in all organs examined, including leaf (Tl), trifoliate leaf (TL), stem (St), root (RO), pod (PO), flower bud (FB), and immature seed (IS). In SDs, the mRNA abundance of *GmRAV* was the highest in trifoliate leaves and the lowest in pods (Fig. 1A).

The diurnal circadian rhythm of *GmRAV* expression was examined by quantitative real-time RT-PCR in trifoliate leaves sampled at 25 d (transferred at 10 d). *GmRAV* transcript exhibited a diurnal circadian rhythm under SDs, suggesting that their expression was partly regulated by circadian clock genes. The expression level of *GmRAV* increased slightly 4 h after dawn, reaching a peak 4 h after the beginning of the dark period and decreased toward dawn, reaching the lowest 4 h before dawn under SDs (Fig. 1B). The amplitude of *GmRAV* mRNA increased significantly under SDs when compared with LDs.

The abundance of the *GmRAV* mRNA in leaves during the shift from SDs to LDs was investigated (Fig. 1B). The time course-dependent expression patterns of *GmRAV* were also analyzed in ‘Dong Nong 47’ plants grown under SDs and LDs using RNAs isolated from trifoliate leaves that were sampled at 4 h after dusk. The levels of *GmRAV* transcripts under SDs were relatively low at 20 DAE but increased sharply to their maximum levels at 23 DAE and thereafter decreased until 45 DAE (the time of flower bud formation) (Fig. 1C). In contrast, under LDs, the transcript levels of *GmRAV* increased slightly at 23 DAE, and thereafter decreased showing lower levels than that under SDs at all the times (Fig. 1C). Overall, the gene expression studies indicated that *GmRAV* was SD-inducible gene in soybean leaves.

In addition, promoter activity of *GmRAV* was measured to determine whether continuous darkness could increase *GmRAV* expression like in SDs (Fig. 1B). In 7-day-old seedlings, *GUS* expression of *GmRAV* promoter via histochemical GUS assay was more predominantly detected under continuous darkness than in continuous light conditions in both cotyledons and hypocotyls (Fig. 1D). These results suggested that *GmRAV* was SD and darkness-inducible gene.

### Effects of GmRAV on Photoperiod Controlling of Flowering Time FT Homologs in GmRAV-i Transgenic Soybean

**Figure 2:**
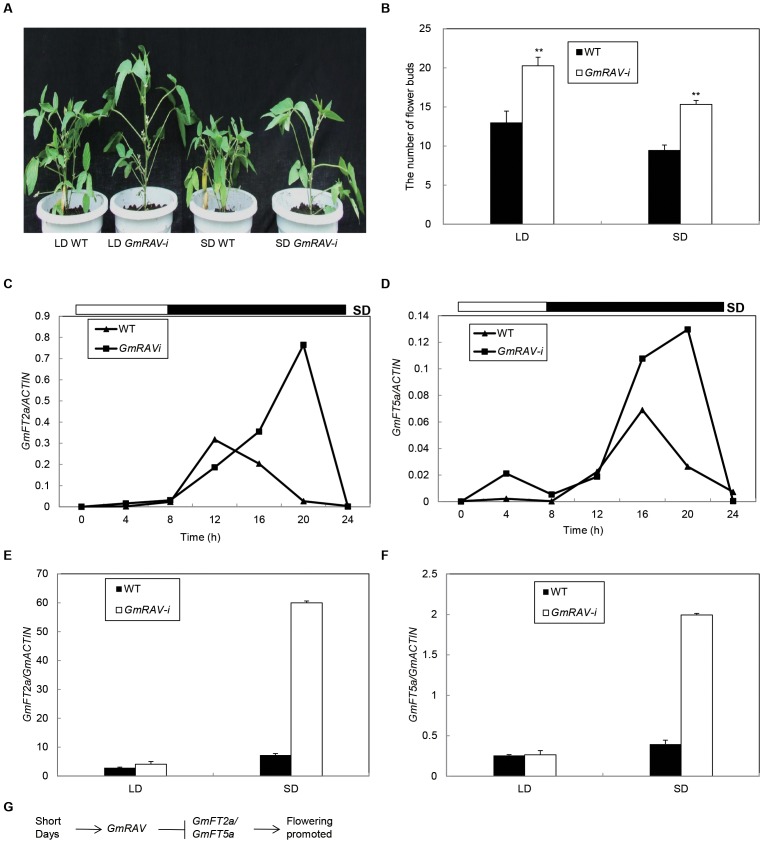
A, Phenotypes of the T6 generation *GmRAV-i* soybean under LDs and SDs. 50-day-old seedlings of WT and *GmRAV-i* transgenic soybean under LDs and SDs at Harbin (planted at May 28). B, The compare of the number of flower buds between WT plants and *GmRAV-i* soybeans under LDs and SDs. The flower buds of 50 plants were measured for each treatment. Error bars represent the SE. **Significant differences in comparison to the non-transgenic lines at P<0.01 (Student’s t test). C and D, Diurnal expression of soybean FT homologs: *GmFT2a* and *GmFT5a* in *GmRAV-i* soybeans grown under SDs (8 h/16 h light/dark). Trifoliate leaves were sampled every 4 h at 15 DAE. White and black bars at the top represent light and dark phases, respectively. Samples were processed and analyzed by RT-PCR as described in Experimental procedures. The levels of *GmACTIN* expression were used as a normalization control, respectively. Average and SE values for three replications are given for each data point. E and F, Relative transcript levels of *GmFT2a* and *GmFT5a* mRNA in *GmRAV-i* soybean leaves under SDs and LDs. Soybean leaves were harvested at 4 h before dawn at 25-day-old under LDs and SDs. G, Pathway controlling flowering in response to short days in soybean.

The transcript abundance of *GmRAV* was affected by day length in soybean leaves, the diurnal phase of *GmRAV* mRNA expression was regulated by the circadian clock, and higher levels of *GmRAV* mRNA were accumulated under SDs than under LDs. To examine whether *GmRAV* acted in a photoperiod functional context, the flowering time in WT and *GmRAV-i* soybeans under SDs and LDs were analyzed. The early-flowering phenotype soybean mutant *GmRAV-i* was observed despite of the day length in both LDs and SDs compared to the WT (Fig. 2A). Apart from their flowering-time phenotype, *GmRAV-i* soybeans also displayed complex pleiotropic alterations of vegetative development. Earlier emergence, reduced numbers of branches and leaves, longer petioles, larger leaves, increased apical dominance, and earlier flowering and maturity were the major phenotypic effects of *GmRAV-i* in all T6 generation plants in both SDs and LDs (Fig. 2A, Table 3).

**Table 3:** Comparison of growth parameters of transgenic T6 *GmRAV-i* soybean plants and wild type (WT) that were under LDs and SDs at Harbin.

		LD WT	LD *GmRAV-i*	SD WT	SD *GmRAV-i*
44 day	Plant height (cm)	25.72	29.62**	20.32	27.63**
	Flower bud number	12.97	20.26**	8.43	16.33**
Maturity stage	Plant height (cm)	65.73	71.13**	53.15	68.23**
	Internode number	19.00	20.33**	15.60	16.00**
	Branch number	4.80	3.67*	4.30	3.33*
	Pod number per plant	85.93	99.33	51.15	80.67
	Seed number per plant	111.47	161.60*	67.34	141.67*
	Seed weight per 100 (g)	6.19	6.47	6.00	6.34

*Differences in comparison to the wild type at 0.01<P<0.05 (Student’s t test),

**Significant differences in comparison to the wild type at P<0.01 (Student’s t test).

In Arabidopsis, *FT* transcript levels oscillated with distinct circadian rhythms (Suárez-López et al., 2001). To check *FT* transcription levels in soybeans, the diurnal circadian rhythm of *FT* gene expression was analyzed by quantitative real-time RT-PCR for *GmFT2a* and *GmFT5a* in trifoliate leaves sampled at 15 d after emergence (DAE) in *GmRAV-i* soybean. The expression level of both *GmFT2a* and *GmFT5a* reached a peak 4 h after the beginning of the dark period and decreased toward dawn in *GmRAV-i* soybean plants under SDs. The amplitude and overall level of *GmFT2a* and *GmFT5a* mRNA were much higher in *GmRAV-i* soybean plants than in WT plants under SDs (Fig. 2C, D). Moreover, the transcript abundance of *GmFT2a* and *GmFT5a* was highly affected in *GmRAV-i* transgenic soybean leaves compared to the wild-type seedlings under SDs than under LDs (Fig. 2E, F). The results indicated that *GmRAV* was a SD-inducible flowering repressor in the flowering response of SD-induced soybeans by repressing positive regulator *GmFT2a* and *GmFT5a* gene expression. This work therefore described the conservation of components and sequence order of a pathway controlling flowering in response to day length. It revealed that the promotion of flowering in short days in *GmRAV-i* soybean resulted from the repression of *GmFT2a* and *GmFT5a* by *GmRAV* (Fig. 2G).

### Effects of GmRAV on the Photoperiod Controlling by Hypocotyl Elongation

**Figure 3:**
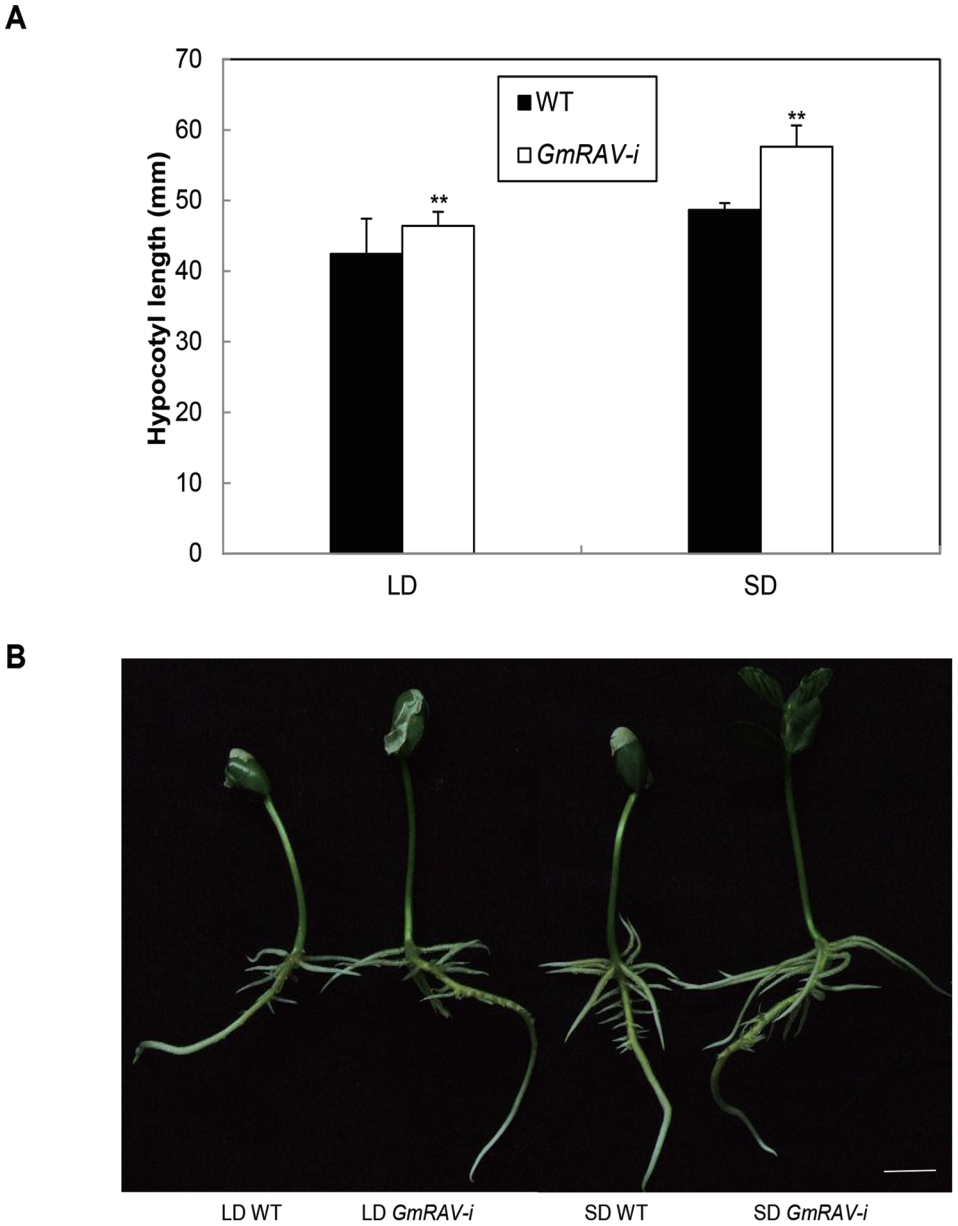
Effects of day length on hypocotyl length in 9-day-old wild-type and *GmRAV-i* soybean seedlings under LDs and SDs. A, Histograms of the mean (*n = *20) for seedlings grown on medium. All seedlings were transgenic for the soybeans indicated. The seedlings were scored 9 d after sowing. Scale bar = 10 mm. **Significant differences in comparison to the non-transgenic lines at P<0.01 (Student’s t test). B, Representative seedlings are shown.

**Figure 4:**
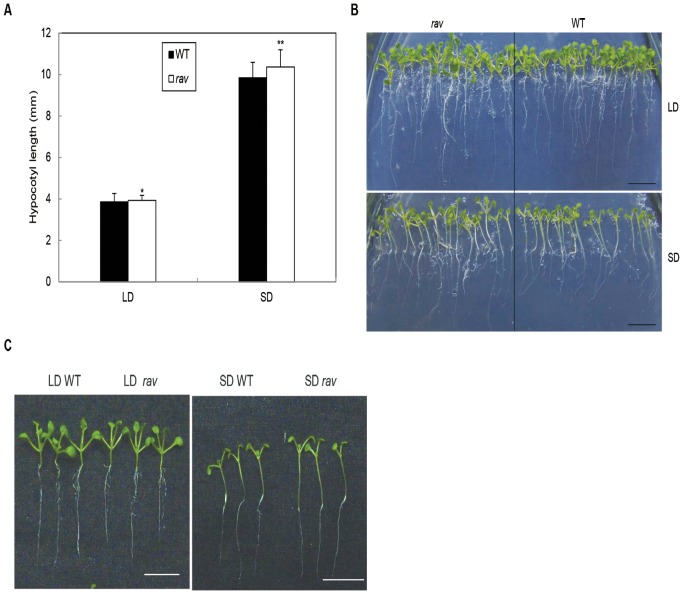
Effects of day length on hypocotyl length in 9-day-old wild-type and Arabidopsis *rav* mutants under LDs and SDs. A, Histograms of the mean (*n = *20) for seedlings grown on medium. The seedlings were scored 9 d after sowing. Scale bar = 10 mm. *differences in comparison to the non-transgenic lines at 0.01<P<0.05, **Significant differences in comparison to the non-transgenic lines at P<0.01 (Student’s t test). B, Phenotype of 9-day-old WT seedlings and Arabidopsis *rav* mutants on MS medium under LDs and SDs. C, Representative seedlings are shown.

To further investigate whether *GmRAV* was SD-inducible, the test of hypocotyl elongation was conducted in LD and SD-grown knock-out soybean mutant *GmRAV-i*, and *Arabidopsis rav* mutants and wild-type plants. *GmRAV-i* soybeans showed SD -mediated hypocotyl elongation responses compared to the wild-type seedlings (Fig. 3A, B). In *GmRAV-i* soybeans, *GmRAV* enhanced SD-mediated hypocotyl elongation response. In comparison, *Arabidopsis rav* mutant displayed the same hypocotyl elongation response to SDs (Fig. 4A). This was evident when grown in LDs or SDs (Fig. 4B, C). These results indicated that *GmRAV* also played a negative role in SD-mediated regulation of hypocotyl elongation.

### Effects of Sucrose on the Flowering of Arabidopsis Rav Mutant

**Figure 5:**
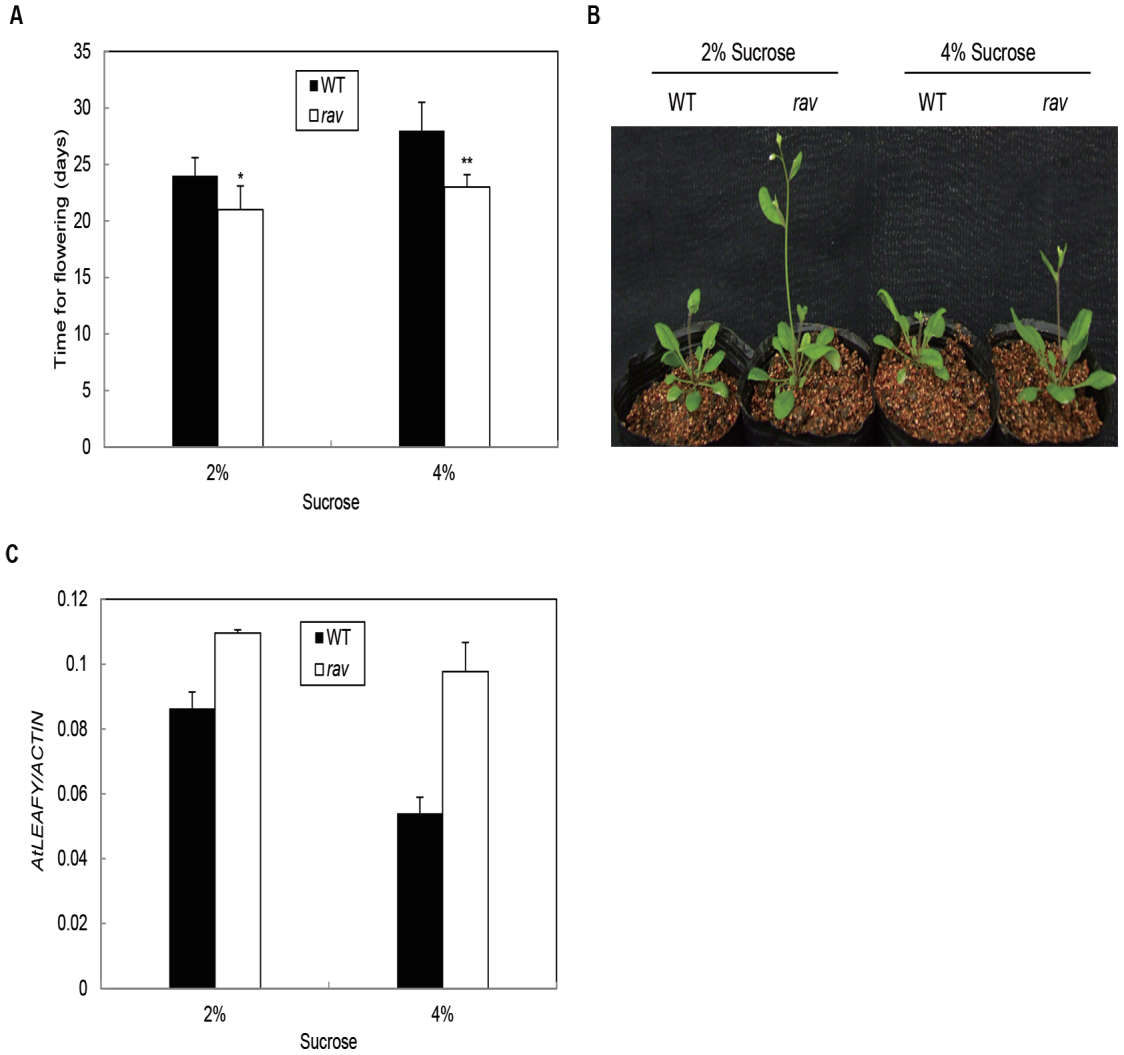
Effects of sucrose on flowering time for Arabidopsis. A, Arabidopsis *rav* mutants and WT seedlings were grown on media with various concentrations of sucrose for 2 weeks, and then transferred to soil under LDs. The flowering time of seedlings with 2% sucrose was WT (24±0.5) and Arabidopsis *rav* mutant (21±0.8). WT plants are the control for Arabidopsis *rav* mutants. Values are the average of 30 to 45 plants. The error bars indicate one SE of the mean. Similar results were obtained in two independent experiments. *Differences in comparison to the non-transgenic lines at 0.01<P<0.05, **Significant differences in comparison to the non-transgenic lines at P<0.01 (Student’s t test). B, Phenotypes of the Arabidopsis *rav* mutant. 23-day old seedlings of WT and Arabidopsis *rav* mutant under natural day length (LD) with treated by 2% and 4% sucrose. C, Quantitative real-time RT–PCR analysis of *LFY* expression in Arabidopsis *rav* mutants. Control amplification of *18 s rRNA* transcript indicated equal amounts of cDNA.

Masa-aki et al. reported that 2 weeks culture was enough to observe the negative effects of high levels of sucrose on floral transition [24]. Therefore, wild-type plants and *Arabidopsis rav* mutant seedlings were grown in culture medium containing 2% (w/v) and 4% (w/v) sucrose for 2 weeks, respectively and then were transferred to soil. Flowering time of Arabidopsis WT and *rav* mutants were examined on MS medium containing 4% sucrose in LDs. In comparison to the plants that were grown on 2% sucrose plates, *rav* mutants showed a 2-day delay in flowering time, whereas WT plants showed 4-day delay (Fig. 5A). These results supported our hypothesis that Arabidopsis *rav* mutants responded to sucrose signaling in plant growth and development. Sucrose affecting flowering time was also observed in the developmental phenotypes (Fig. 5B). Overall, these results indicated that *AtRAV* participated in the regulation of high levels of sucrose-dependent flowering response.

To investigate the reason why high concentration sucrose could delay flowering, different levels of *FT*, *SOC1*/*AGL20* and *LEAFY* (*LFY*) expressions were analyzed by reverse transcriptase (RT)-PCR in Arabidopsis WT plants and *rav* mutants grown respectively on media with 2% and 4% sucrose for 15 d. Both expression levels of *FT* and *SOC1*/*AGL20* in Arabidopsis *rav* mutants as well as WT plants on media with 2% (w/v) were identical with on media with 4% (w/v) sucrose under LD conditions (data not shown). *LFY* was expressed in the leaf primordium before the transition to flowering. In this study, *LFY* expression levels were reduced under the supplementary of 4% (w/v) sucrose compared to 2% (w/v) sucrose (Fig. 5C). The results suggested that increased concentration of sucrose could lead to decrease the expression of *LFY* gene. It also showed that high concentration of sucrose in growth media delayed the flowering time in Arabidopsis. Moreover, transcript level of *LFY* was greatly reduced in *rav* mutants than in WT plants on media containing high level of sucrose under LDs. The results displayed that *GmRAV* delayed flowering in high level of sucrose by regulating the expression of *LFY*.

### The Responses of Arabidopsis Rav Mutant, GmRAV-ox Tobacco and GmRAV-i Soybean Seedlings to Exogenous Sucrose

**Figure 6:**
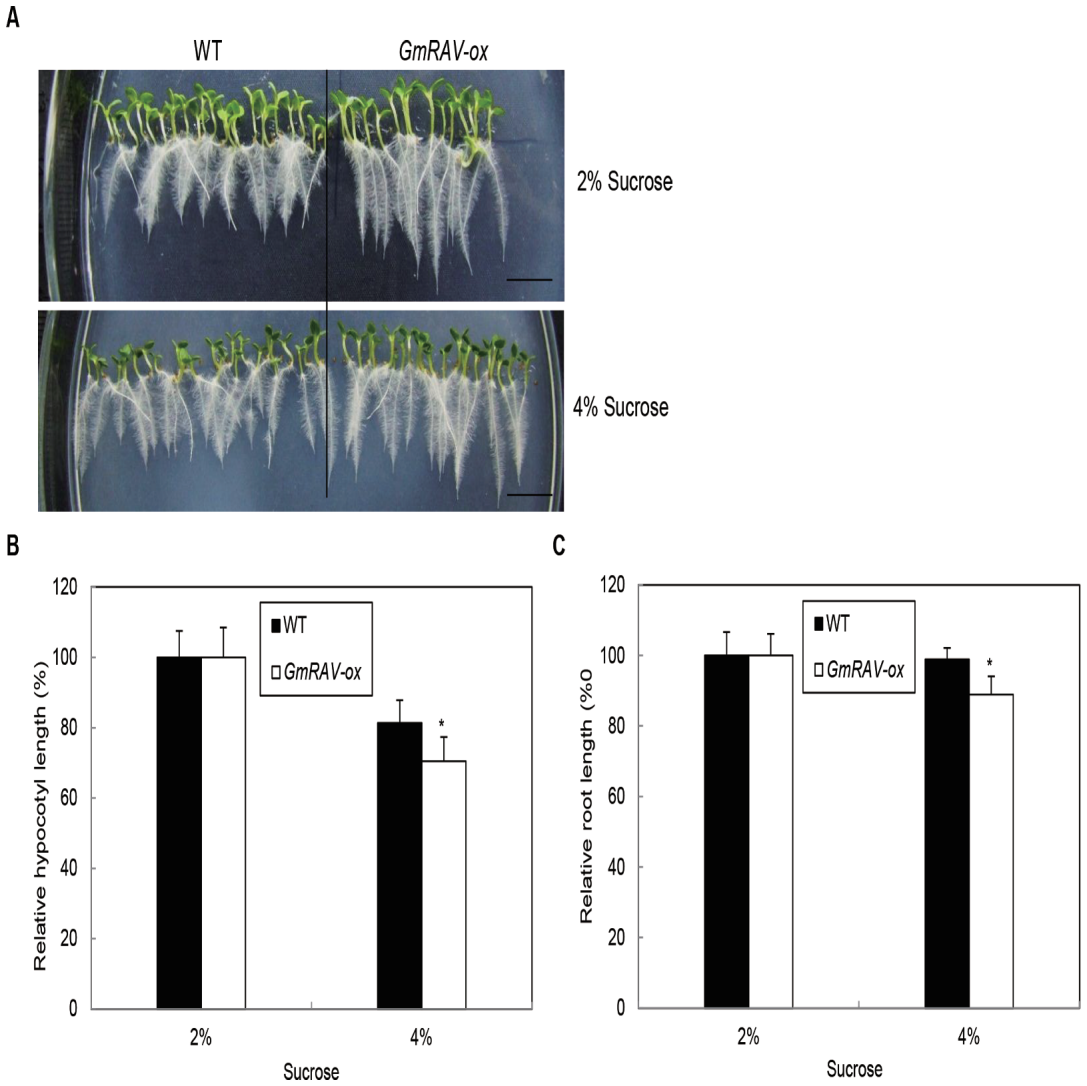
Response of wild-type and *GmRAV-ox* tobacco seedling to sucrose. A and B, Relative hypocotyl and root growth in response to various concentrations of sucrose. The length of hypocotyl of tobacco seedlings grown 2% (w/v) sucrose was 4.91±0.75 mm for the WT and 5.85±0.76 mm for *GmRAV-ox*. Root length, of seedlings grown 2% (w/v) sucrose was 15.8±1.21 mm in the WT and 18.8±1.94 mm in *GmRAV-ox*. The hypocotyl and root length of 20–30 seedlings were measured for each treatment. Error bars represent the SE. C, Phenotype of 7-day-old WT seedlings and T3 generation *GmRAV-ox* tobaccos on MS medium containing 2% and 4% sucrose. *Differences in comparison to the non-transgenic lines at 0.01<P<0.05 (Student’s t test).

**Figure 7:**
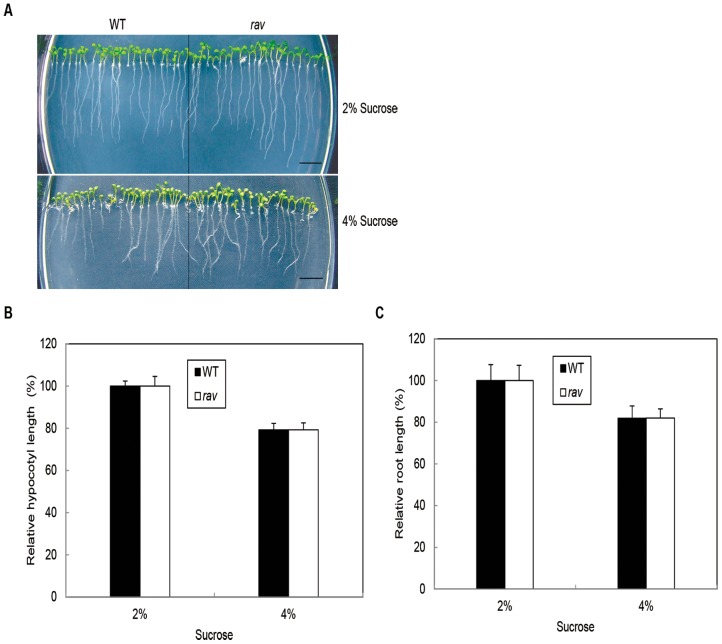
Response of WT and Arabidopsis *rav* mutants to sucrose on development. A and B, Relative hypocotyl and root growth in response to various concentrations of sucrose. Hypocotyl length, as a percentage of the untreated control, of seedlings grown on sucrose. The length of hypocotyl of Arabidopsis seedlings grown 2% (w/v) sucrose was 4.26±0.24 mm for the WT and 4.06±0.46 mm for Arabidopsis *rav* mutant. Root length, as a percentage of the untreated control, of seedlings grown on sucrose. Root length, of seedlings grown without sucrose was 26.70±1.16 mm in the WT and 28.24±1.33 mm in Arabidopsis *rav* mutant. The hypocotyl and root length of 20–30 seedlings were measured for each treatment. Error bars represent the SE. C, Phenotype of 7-day-old WT seedlings and Arabidopsis *rav* mutants on MS medium containing 2% and 4% sucrose.

**Figure 8:**
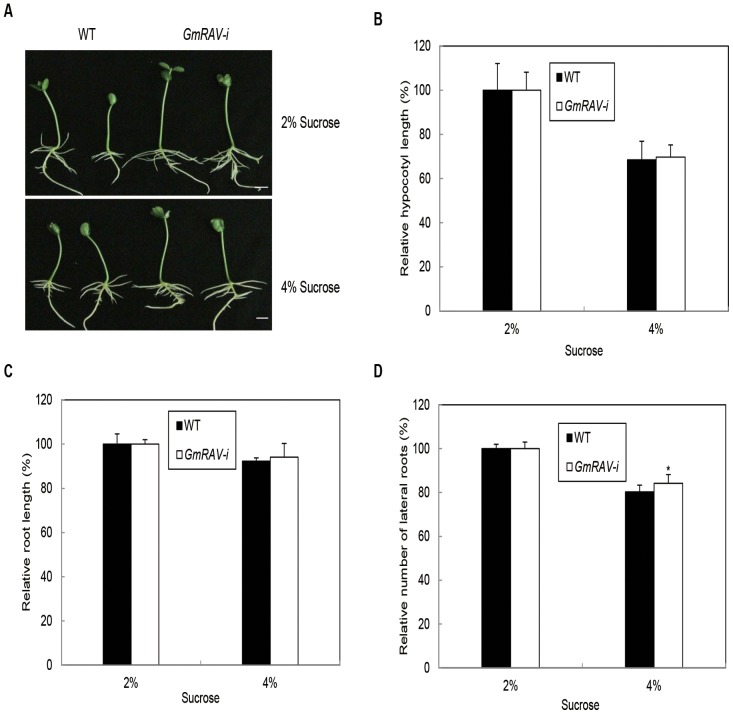
Response of WT and *GmRAV-i* soybean seedlings to sucrose. A and B, Relative hypocotyl and root growth in response to various concentrations of sucrose. Hypocotyl length, as a percentage of the untreated control, of seedlings grown on sucrose. The length of hypocotyl of soybean seedlings grown without sucrose or glucose was 57.17±1.30 mm for the WT and 61.17±1.97 mm for *GmRAV-i* soybean. Root length, as a percentage of the untreated control, of seedlings grown on sucrose. Root length, of seedlings grown without sucrose or glucose was 60.33±1.30 mm in the WT and 65.67±1.97 mm in *GmRAV-i*. Error bars represent the SE. C, Relative number of lateral roots in response to various concentrations of sucrose. The number of lateral roots, as a percentage of the untreated control, of seedlings grown on sucrose. Error bars represent the SE. The number of lateral roots of seedlings grown without sucrose and glucose was 17.83±0.75 for the WT and 21.17±0.98 for *GmRAV-i* soybean. The number of lateral roots of 20–30 seedlings was scored for each treatment. *Differences in comparison to the non-transgenic lines at 0.01<P<0.05. D, Phenotypes of 7-day-old WT and *GmRAV-i* soybean seedlings on MS medium containing 2% and 4% sucrose. *Differences in comparison to the non-transgenic lines at 0.01<P<0.05 (Student’s t test).

To analyze the possible function of *GmRAV* in response to sucrose stress, we studied the responses to exogenous sucrose application using Arabidopsis *rav*, *GmRAV-ox* tobacco, and *GmRAV-i* soybean plants. Sensitivity of *Arabidopsis rav* mutants and *GmRAV-ox* tobacco seedlings in response to sucrose was tested in root and hypocotyl by growth inhibition assays. The hypocotyl and root lengths of *GmRAV-ox* tobacco and WT plants were inhibited by both concentrations of sucrose (2% and 4%) (Fig. 6A), but *GmRAV-ox* tobacco were more remarkably reduced compared to WT plants (Fig. 6B, C). These results suggested that *GmRAV-ox* tobacco were more sensitive to sucrose than WT plants in the assays of hypocotyls and roots growth inhibition. Fig. 7A showed that the hypocotyl and root lengths of Arabidopsis *rav* mutants and WT plants were inhibited by both concentrations of sucrose. However, inhibited extent of *rav* mutants and WT plants by sucrose in hypocotyl and root inhibition assays was identical (Fig. 7B, C). Likewise, the growth in soybean *GmRAV-i* and WT plants in terms of hypocotyl lengths, main root length and the number of lateral roots was all inhibited by both concentrations of sucrose (Fig. 8A). Hypocotyl, root length, and the number of lateral roots of *GmRAV-i* soybeans were less inhibited by sucrose than in WT plants (Fig. 8 B–D). Therefore, *GmRAV-i* seedlings exhibited insensitive phenotypes, as compared with the WT plant, during sucrose-mediated root and hypocotyl growth inhibition. Overall, in agreement with the *GmRAV-ox* tobacco results, the Arabidopsis *rav* mutants and the *GmRAV-i* soybean seedlings showed a significantly decreased sensitivity to sucrose by root and hypocotyl growth inhibition assays.

**Figure 9:**
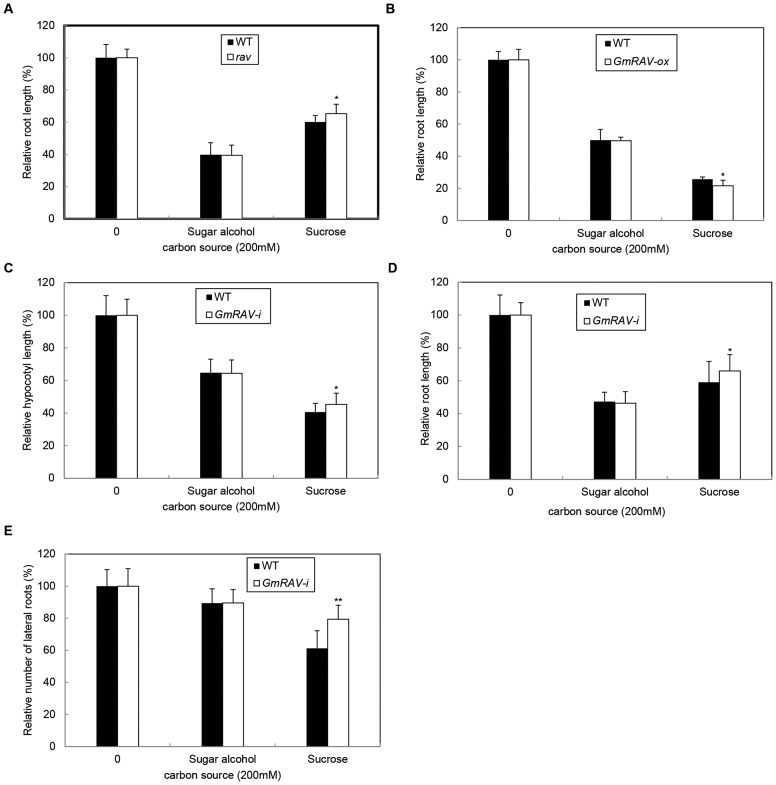
Response of the WT plants, Arabidopsis *rav* mutants and *GmRAV-ox* tobaccos or *GmRAV-i* soybean seedlings to full strength MS media supplemented with the indicated sugar concentration. A and B, Relative root length in response to 200-day-old Arabidopsis and tobaccos. Root length, as a percentage of the untreated control, of seedlings grown on carbon source. Error bars represent the SE. Root length, of seedlings grown without carbon sucrose was WT (15.96±1.2 mm) and Arabidopsis *rav* mutant (19.25±0.8 mm), and for tobaccos without carbon sucrose was WT (15.29±0.9 mm) and *GmRAV-ox* (18.4±1.2 mm). Root length of 20–30 seedlings was measured for each treatment. C and D, Relative hypocotyl and root length in response to 200 mM carbon source in 8-day-old soybeans. Hypocotyl and root length, as a percentage of the untreated control, of seedlings grown on carbon source. Error bars represent the SE. Hypocotyl length without carbon source was WT (50.67±1.2 mm) and *GmRAV-i* (54.83±0.9 mm), and main root length was WT (67±1.2 mm) and *GmRAV-i* (79.83±0.7 mm). E, Relative number of lateral roots in response to 200 mM carbon source. Number of lateral roots, as a percentage of the untreated control, of seedlings grown on carbon source. Error bars represent the SE. The number of lateral roots, of seedlings grown without carbon source was WT (16.33±0.5) and *GmRAV-i* (21±1.0). Number of lateral roots of 20–30 seedlings was scored for each treatment. *differences in comparison to the non-transgenic lines at 0.01<P<0.05, **Significant differences in comparison to the non-transgenic lines at P<0.01 (Student’s t test).

The addition of chemicals to a medium changes both the chemical composition and the osmotic potential of medium. To identify whether the negative effects of sucrose on development and flowering were due to metabolic or osmotic factors, we examined the effects of both mannitol and sorbitol. These two sugar alcohols were widely used as the osmotic controls in plant. Therefore, a combined osmotic control value was calculated from both mannitol- and sorbitol-grown plants. Sugar effects were compared to the combined osmotic control value. Plants grown in the absence of any supplemental carbon (sugar alcohol or sucrose) were also measured for each parameter in order to observe the overall effect of increasing osmolarity. Increasing osmolarity of the growth media by sugar alcohols inhibited the plant growth when compared to untreated media. The behavior of Arabidopsis *rav* mutant, *GmRAV-ox* tobacco and *GmRAV-i* soybean seedlings with 200 mM sugar alcohols on MS media showed no difference from that of the WT seedlings in hypocotyl, root and the number of lateral roots growth inhibition assay (Fig. 9 A–E). However, *rav* mutants and soybean *GmRAV-i* in terms of hypocotyl lengths, main root length and the number of lateral roots of development that exhibited insensitive phenotypes were less inhibited by 200 mM sugars (6.8%) (Fig. 9A, C–E), which indicated *RAV* gene played a negative role in sucrose-mediated regulation of hypocotyl and root elongation due to metabolic effects rather than osmotic effects.

## Discussion

### GmRAV is a Novel Negative Regulator of SD-mediated Flowering and Hypocotyl Elongation

The time of flowering induction determines to a large extent the reproductive success of plants. Plants integrate diverse environmental and endogenous signals to ensure the timely transition from vegetative to flowering period. In many plant species, floral transition is strongly controlled by the circadian clock. The clock with a period close to 24 h serves to coordinate diurnal rhythms with physiology and behavior. GmRAV belongs to the RAV protein family containing two domains: the AP2 and the B3 DNA-binding domain. Given that long-day plant Arabidopsis *TEM1, TEM2* and chestnut *CsRAV1* genes were circadian regulated [35], [36], we examined the possibility that soybean gene was rhythmically expressed in short-day plant soybean leaves. Higher level of *GmRAV* transcripts was accumulated in soybean leaves in SDs than in LDs which was almost suppressed, and it was also regulated by the circadian clock. The *GmRAV* mRNA reached a peak 4 h after the beginning of the dark period in SDs, whereas *CsRAV1* mRNA peaked at noon, *TEM1* and *TEM2* peaked at dusk in LDs. This different time of expression in day suggests that although *RAV* gene shows high homology among different day-length plants, it may play different roles in different day lengths. Furthermore, the time course-dependent expression pattern of *GmRAV* was analyzed in ‘Dong Nong 47’ plants grown under SDs and LDs, and showed that the level of *GmRAV* transcript in SDs was higher than in LDs till flowering, confirming that *GmRAV* was SD-inducible gene.

We further analyzed the function of repressing flowering of *GmRAV* gene based on the earlier flowering phenotypes of *GmRAV-i* soybeans than WT under both SDs and LDs. In Arabidopsis, *TEM1 and TEM2* also acted as direct *FT* repressors and repressed flowering under LD and SD conditions [35], [37]. Likewisely, the mRNA levels of *GmFT2a* and *GmFT5a* were also examined in WT and *GmRAV-i* soybeans under SDs, and they were evidently enhanced in earlier flowering *GmRAV-i* soybeans, which indicated that *GmRAV* played a negative role by repressing *FT* genes in the SD-mediated photoperiod control of flowering in soybean, whereas in Arabidopsis, *TEM1* repressed flowering by repressing *FT* genes in LDs [35]. We speculated that a genetic pathway similar to that in Arabidopsis was conserved in the photoperiod control of flowering in soybean, a SD plant.

Furthermore, in photoperiod control of hypocotyl elongation assays, both *GmRAV-i* soybeans and Arabidopsis *rav* mutants were hypersensitive in SD-mediated promotion of hypocotyl elongation. Therefore, we concluded that the *RAV* role was also conserved in the photoperiod control of hypocotyl elongation in soybean and Arabidopsis.

### GmRAV Affected Development and Flowering Time in the Presence of Exogenous Sucrose

Carbohydrates are thought to play a crucial role in the regulation of flowering. The relation between sugar metabolism/signaling and floral transition received extensive attention lately [25]. Sugar signaling was of great importance in flowering time control, which directly affected yield [38], [39]. The work of Heyer et al. already provided clear evidence that flowering time control is strongly influenced by modifying sugar balances in the apex [40]. Several sucrose signaling insensitive Arabidopsis mutants have been identified based on the effect of high levels of external sugars on seedling growth and development such as *cai* (carbohydrate insensitive) [41], *isi* (impaired sugar induction) [42], *lba* (low levels of β-amylase) [43], *rsr* (reduced sugar response) [44], *sis* (sugar insensitive) [45], *sun* (sucrose uncoupled) mutants [46]. For example, *sig* (sucrose insensitive growth) mutant was selected on media containing 350 mM sucrose [47]. Similarly, Arabidopsis *rav* mutants and soybean *GmRAV-i* in terms of hypocotyl lengths, main root length and the number of lateral roots of development which exhibited insensitive phenotypes were less inhibited by 200 mM sugars.

To ensure uniform sugar responses, the initial mutant isolation screens were mostly performed on media containing high sucrose concentrations, raising concerns about the physiological relevance of sugar regulation. Moreover, the phenotypes could also be influenced by osmotic stress in the media. In this study, we treated Arabidopsis *rav* mutants, *GmRAV-ox* tobaccos and *GmRAV-i* soybeans with 200 mM supplemental carbon sources, and observed that transgenic plants and WT were almost uniformly inhibited by only supplemental sugar alcohols. A high concentration of sucrose added into basal medium also inhibited development both WT and transgenic plants. But we found the behavior of Arabidopsis *rav* mutant, *GmRAV-ox* tobacco and *GmRAV-i* soybean seedlings with 200 mM sugar alcohols on MS media showed no difference from that of the WT seedlings in hypocotyl, root and the number of lateral roots growth. Thus, the specific effects of sucrose treatment could be attributed to the chemistry of the sucrose itself rather than to osmotic effects of the sucrose, which indicated *RAV* gene played a negative role in sucrose signaling due to metabolic effects rather than osmotic effects.

In Arabidopsis, the delay in flowering time caused by high concentrations of glucose in media was previously reported [23]. Sugar seemed to affect a specific part of the vegetative phase, rather than all phases [24]. Flowering time of Arabidopsis *rav* mutants were less reduced than WT under high concentration of 4% sucrose condition, which showed Arabidopsis *rav* mutants were also insensitive to sucrose in flowering time. Moreover, the expression of *LFY* was much more down-regulated in *Arabidopsis* WT plants compared to *rav* cultivated in high concentrations of sucrose, indicating that *RAV* might be also a positive regulated factor in flowering time inhibition assays.

In summary, as an important process in plant reproduction, flowering time is finely controlled by complex network. *GmRAV* was both involved in negative regulation of the photoperiodic control of flowering time responses and positive regulation of sucrose control of flowering time.
